# A CRISPR/Cas12a-based DNAzyme visualization system for rapid, non-electrically dependent detection of *Bacillus anthracis*

**DOI:** 10.1080/22221751.2021.2012091

**Published:** 2022-02-01

**Authors:** Dongshu Wang, Gang Chen, Yufei Lyu, Erling Feng, Li Zhu, Chao Pan, Weicai Zhang, Xiankai Liu, Hengliang Wang

**Affiliations:** State Key Laboratory of Pathogen and Biosecurity, Beijing Institute of Biotechnology, Beijing, People’s Republic of China

**Keywords:** CRISPR-Cas12a, DNAzyme, *Bacillus anthracis*, detection, colour change

## Abstract

As next-generation pathogen detection methods, CRISPR-Cas-based detection methods can perform single-nucleotide polymorphism (SNP) level detection with high sensitivity and good specificity. They do not require any particular equipment, which opens up new possibilities for the accurate detection and identification of *Bacillus anthracis.* In this study, we developed a complete detection system for *B. anthracis* based on Cas12a. We used two chromosomally located SNP targets and two plasmid targets to identify *B. anthracis* with high accuracy. The CR5 target is completely new. The entire detection process can be completed within 90 min without electrical power and with single-copy level sensitivity. We also developed an unaided-eye visualization system based on G4-DNAzyme for use with our CRISPR-Cas12a detection system. This visualization system has good prospects for deployment in field-based point-of-care detection. We used the antisense nucleic acid CatG4R as the detection probe, which showed stronger resistance to interference from components of the solution. CatG4R can also be designed as an RNA molecule for adaptation to Cas13a detection, thereby broadening the scope of the detection system.

## Introduction

Anthrax, a serious zoonotic disease, is caused by *Bacillus anthracis*. Because *B. anthracis* spores can be used as biological warfare and terrorism agents, the development of sensitive, efficient detection methods for this pathogen is important and urgently needed.

Currently, *B. anthracis* is detected by identifying its vegetative cells and spores, toxin and specific antigens, or nucleic acids. Conventional microbiological methods used to detect vegetative cells and spores of this pathogen rely on characteristic phenotyping approaches. Although such methods are usually considered the gold standards for *B. anthracis* detection, many of the phenotypic features of *B. anthracis* are shared by other *B. cereus* group isolates [1,2]. Traditional methods must therefore include bacterial culturing which makes them unsuitable for rapid detection of *B. anthracis*. Anthrax toxin detection is currently the most widely used antigen-based system, but approaches that rely on various other antigens (e.g. the BclA exosporium glycoprotein) [3]. ELISA-based methods are the most commonly used, but techniques such as the Luminex assay [4], the magnetic particle fluorogenic immunoassay [5], lateral flow assays [6], and biosensors [7,8] have been investigated as well. Although greatly improved by high-precision instruments, the sensitivity of antigen-based methods is often insufficient, and cross-reactivity with other *B. cereus* group members still cannot be completely avoided. Nucleic acid-based *B. anthracis* detection has many advantages, including no need to cultivate microorganisms as well as the ability to use inactivated samples. *B. anthracis* can be detected with a wide variety of DNA amplification-based methods, including polymerase chain reaction (PCR), real-time PCR (RT–PCR), and isothermal amplification techniques*.* The most important issue when using these methods, which are usually rapid and sensitive, is the selection of a species-specific nucleic acid fragment. Because of the high degree of genetic similarity between *B. anthracis* and other *B. cereus* group members [9,10], however, achieving high specificity is not a simple task. As with antigen-based methods, the most commonly used targets are the genes encoding anthrax toxins and capsular proteins. These genes are located on pXO1 and pXO2 virulence plasmids; therefore, strains lacking these plasmids will test negative for *B. anthracis*. Non-plasmid-based targets have also been selected for use, but more isolates are needed to verify their reliability. For example, BA813, which was initially recognized as a specific target in *B. anthrac*is, was subsequently found in several *B. cereus* and *B. thuringiensis* strains [11,12]. Although whole genome sequencing provides the most accurate results, the interpretation of large amounts of sequencing data is not easy. Even the GenBank database contains individual *B. cereus* genomes that have been incorrectly assigned to *B. anthracis*. The main requirement for nucleic acid-based detection of *B. anthracis* is the ability to differentiate genetically similar strains. First, an accurate detection target is needed-usually a conserved single-nucleotide polymorphism (SNP) site[9]. Second, a method that can distinguish SNP differences must be used. Nevertheless, the detection of SNPs in the field by first-line responders in cases of suspected anthrax release is challenging. Detection methods based on qPCR (quantitative Real-time PCR) and sequencing are thus widely applied, but these techniques require expensive equipment and experienced operators. CRISPR-based methods are a better choice for on-site detection.

CRISPR/Cas-based detection methods are considered to be next-generation pathogen detection techniques [13]. The most widely used CRISPR-associated protein (Cas) types used for nucleic acid detection are Cas9, Cas12, Cas13, and Cas14b [13,14]. In 2017, Gootenberg et al. [15] developed a Cas13a-based *in vitro* nucleic acid detection method, which they named SHERLOCK (for specific high-sensitivity enzymatic reporter unlocking). In this system, Cas13a binds to the target RNA under the guidance of crRNA. “Collateral-cleavage” activity is then activated to non-specifically degrade a fluorescently labelled RNA reporter, which in turn releases a fluorescence signal that indicates the presence of the target nucleic acid. Two additional methods that have been developed, DETECTR (DNA endonuclease targeted CRISPR trans-reporter) [16] and HOLMES (one-HOur Low-cost Multipurpose highly Efficient System) [17], can detect DNA sequences with attomolar sensitivity and high specificity based on the trans-cleavage activities of CRISPR–Cas12a [18]. The first signal reporting system for CRISPR-based detection involved a fluorescent probe, with the trans-cleavage activity of the Cas protein used to cleave the fluorescence-quenching probe to generate a fluorescence signal. Electrochemical sensors have also been used in conjunction with the CRISPR method. The most direct application is the use of nucleic acid probes to connect electrochemically active substances to electrodes to generate a strong electrical signal response after cleavage. Among them, LFD has been used in the SHERLOCK v2 system, which is very convenient for point-of-care testing (POCT). However, it needs to carefully control the probe volume in an appropriate range.

CRISPR/Cas-based detection methods can identify SNPs with high sensitivity and good specificity, and the lack of specific equipment requirements with these methods provides new possibilities for detecting and identifying *B. anthracis.* Because of their inherent catalytic activities, ease of use, and high sensitivities, DNAzyme-based biosensors have been widely exploited for bioanalysis. In this study, we used recombinase polymerase amplification (RPA) combined with CRISPR–Cas12a to simultaneously detect two plasmid targets and two specific chromosomally located SNP targets. No special equipment was required at any point in this process, and the results were visible to the unaided eye. We also developed a new DNAzyme-based visualization method for this Cas12a detection system, thereby opening up more possibilities for CRISPR detection technologies.

## Materials and methods

### Strains and detection targets

To avoid biosecurity risks, we used the following attenuated plasmid-cured strains: A16Q1 (pXO1-, pXO2+) [19] and A16PI2 (pXO1+, pXO2-) [20]. *Bacillus cereus* BC307 (CMCC 63317; GenBank assembly accession no. GCA_009799965.1) was used as a negative control to confirm detection specificity. Sterile water was used as a blank control. *Bacillus cereus* HN001 (GenBank assembly accession no. GCA_001635995.1), *B. thuringiensis* LM1212 (GCA_000500585.1), *B. subtilis* str. 168 (GCA_000009045.1), and *Staphylococcus aureus* ATCC49521 and DH5α were used in specificity tests. Genomic DNA concentration and copy number quantification are shown in the Supplementary file, Table S1.

The virulence plasmid is the main detection target in *B. anthracis* [21]. The *lef* gene on pXO1 was detected in strain A16PI2, and the *capB* gene on pXO2 was detected in strain A16Q1. In *B. anthracis*, PlcR [22] has been rendered non-functional by a nonsense mutation in the *plcR* gene, a known species-specific marker. This nonsense mutation has been confirmed to be an excellent target for distinguishing *B. anthracis* from its nearest genetic neighbours [23,24]. For use as a target, we selected the CR5 locus from predicted *B. anthracis* CRISPR sites because this region is highly conserved in *B. anthracis* according to sequencing and GenBank verification of 181 Chinese isolates [25].

### Design of PCR primers and crRNAs

The *lef* and *capB* genes located on the *B. anthracis* virulence plasmid are not usually present in closely related species such as *B. cereus* and *B. thuringiensis*. In theory, the 17 bases after the TTTN/TTN protospacer adjacent motif (PAM) sequence [16] in *lef* and *capB* should be usable as target sites for species detection. Five target sequences in each gene were selected to compare the efficiency and specificity of the crRNA (Supplementary file, Table S2).

Two species-specific SNP sites were identified. The first SNP was a CAG→TAG nonsense mutation in the *plcR* gene of *B. anthracis* compared with *B. cereus* BC307. At the second specific SNP site in CR5, a “G” nucleotide was present in *B. anthracis*, whereas a “T” was present in other *B. cereus* group members (Supplementary file, Figure S1).

**Figure 1.**
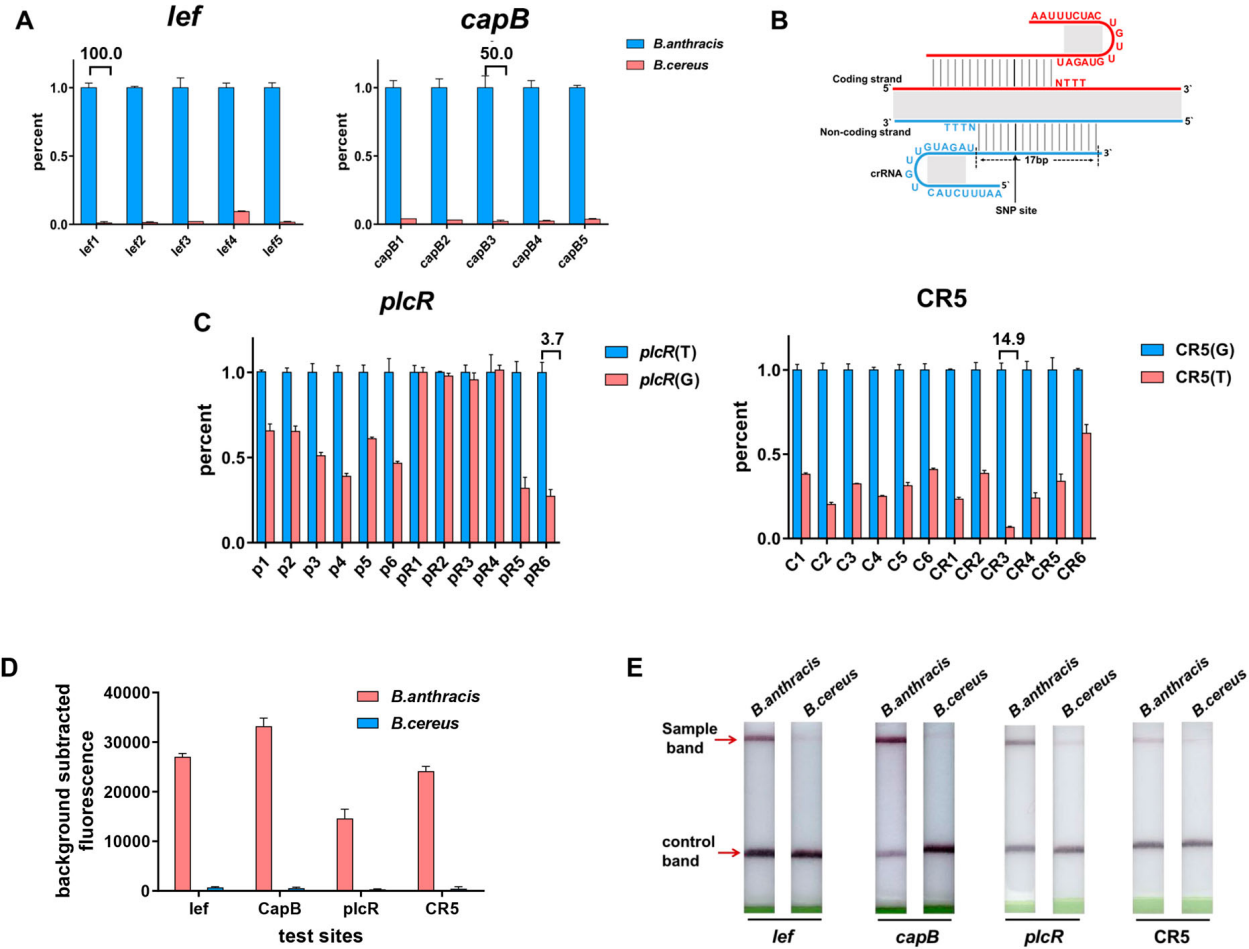
**Screening and optimization of crRNAs for *B. anthracis* detection. (A)** Screening and optimization of crRNAs for *B. anthracis lef* and *capB* gene targeting. Five target sequences containing The ratios of the fluorescence values vs. the average positive values for *B. anthracis* was shown in histograms*.* By comparing the signal-to-noise ratio, the crRNA-lef1 (S/N= 100.00) and crRNA-capB3 (S/N= 50.00) were selected as the best crRNAs for subsequent detection (three technical replicates; bars represent the mean ± SD). (**B)** Design of the crRNAs for SNP targeting. The spacing between the PAM sequence and the SNP site is generally 0–5 bases, and both the coding and non-coding strands are targetable by the crRNAs. **(C)** All 12 possible crRNAs were screened for SNPs in *plcR* and CR5 (three technical replicates; bars represent the mean ± SD). The crRNA-pR6 (S/N= 3.68) and crRNA-CR3 (S/N= 14.93) were selected as the best crRNAs for subsequent detection. **(D)** Histogram indicating relative fluorescent unit (RFU) detection values, with *B. anthracis* and *B. cereus* clearly distinguishable (three technical replicates; bars represent the mean ± SD). All *B. anthracis* detection targets emitted strong fluorescence, whereas *B. cereus* (the negative control) did not. **(E)** LFD strips detection showed all *B. anthracis* detection targets showed clear positive bands, but very weak bands were associated with *B. cereus*.

Because the target nucleotide at the SNP site is probably fixed, a suitable PAM sequence may not be present nearby. Therefore, the ability to detect an SNP site requires the introduction of a PAM sequence using PCR primers. The spacing between the PAM sequence and the SNP site is generally 0–5 bases, and both coding and non-coding strands can be targeted by the crRNA (Figure 1B). Hence, all 12 possible crRNAs were screened to detect SNP sites (Supplementary file, Table S2).

The crRNAs consisted of a 19-nucleotide universal sequence (5′-AAUUUCUACUGUUGUAGAU-3′) [26] located 17 nucleotides downstream of the PAM sequence. The fluorescent probe, a single-stranded DNA probe modified by luminescence and quenching groups, was 12 bases long with a HEX label at the 5′ end and a BHQ1 label at the 3′ end (HEX-5′-GAGACCGACCTG-3′-BHQ1) [17].

### crRNA screening and optimization

The *capB* gene was detected in the A16Q1 strain, and *lef, plcR*, and CR5 loci were detected in the A16PI2 strain. *Bacillus cereus* strain BC307 was used as a negative control. Bacterial genomes were diluted to 20 ng/μL and used as a PCR amplification template with Takara *ExTaq* (version 2.0 kit). PCR amplifications were conducted in 50-mL volumes containing 19 μL double distilled water, 25 μL *ExTaq* mix, 10 μmol/L of each primer, and 2 μL of the diluted template. The PCR cycling protocol was as follows: initial denaturation at 96°C for 5 min, followed by 30 rounds of denaturation at 96°C for 30 s, annealing at 55°C for 30 s, and extension at 72°C for 1 min, with a final extension of 72°C for 5 min.

A 2-μL aliquot of unpurified PCR product was added to the CRISPR–Cas12a reaction system (Supplementary file, Table S3). When the Cas12a protein, crRNA, and PCR amplification products were combined in a ternary complex, the trans-cleaving activity of Cas12a was activated, which allowed cleavage of the labelled single-stranded DNA probe and consequent fluorescence emission. The fluorescence intensity was measured on a Bio-Rad real-time PCR CFX96 instrument (Life Science, Hercules, CA, USA).

### Detection based on RPA and the CRISPR-Cas12a system

To shorten detection times and eliminate reliance on electrically powered devices, traditional PCR methods were replaced by RPA. RPA primers were designed according to the instructions in the assay design manual of a TwistAmp DNA Amplification kit (TwistDx, Maidenhead, UK). To improve the amplification speed and sensitivity of the reaction, the intended length of the amplified product was designed to range from 90 to 150 bp. GC contents and lengths of designed primers were 20%–60% and 32–38 nucleotides, respectively, with no obvious complementary sequences or palindromes present. The RPA primers and the reaction system are detailed in Table S3. To determine the detection threshold, genomic DNA was diluted from 1.5 × 10^3^ aM to 1.5 × 10^−5^ aM. For the detection of *B. anthracis*, vegetative cells and spores were diluted from 10^7^ to 10^0^ CFU/mL. CRISPR–Cas12a reaction conditions were the same as described above.

### Visualization of detection results

Lateral flow dipsticks (LFDs; TwistDx) were used to display the detection results. Single-stranded 12-nucleotide DNA probes were modified with FITC and biotin sequences (FITC-5′-GAGACCGACCTG-3′-biotin) as described above. The Cas12a reaction product (10 μL) was added dropwise to an LFD strip (5 min). The strip was incubated with 100 μL of HybriDetect (TwistDx) assay buffer for 5 min, and the result was immediately observed.

The visualization system, which was based on the peroxidase activity of the G-quadruplex-hemin DNAzyme, was newly developed in this study. DNAzyme CatG4 (whose sequence is 5′-TGGGTAGGGCGGGTTGGGAAA-3′) was selected for the colour reaction. The Cas12a reaction was performed according to the above-described method with the reverse complementary sequence CatG4R (5′-TTTCCCAACCCGCCCTACCCA-3′) added to the Cas12a detection system as a reporter probe. A 20-μL aliquot of the above-described Cas12a reaction product and 2 µL CatG4 (10 μM) solution were mixed, kept in a water bath at 95°C for 5 min, and then cooled to room temperature to anneal the DNAzyme (CatG4) and probe (CatG4R). Next, 2 μL of hemin (100 μM; Sangon Biotech, Shanghai, China) and 72 μL of MES buffer (0.1 M, pH 4.7; Bioroyee Biotechnology, Beijing, China) were added to the annealed solution. After thorough incorporation of 2 μL of 2,2′-azino-di(3-ethylbenzthiazoline-6-sulfonic acid (50 mM; Roche Diagnostics, IN, USA) into the solution, 2 μL H_2_O_2_ (3% [v/v]; Sangon Biotech) was added for colour development. An obvious green colour after 5 min indicated the presence of the target DNA, whereas solutions negative for target DNA were colourless or light green.

## Results

### Screening and optimization of crRNAs for *B. anthracis* detection

To obtain an optimal crRNA, five target sites containing PAM sequences were designed for use with *lef* and *capB* genes. Three independent replicates were performed for each reaction. The crRNA giving rise to the highest fluorescence signal-to-noise ratio was expected to have the best specificity. On this basis, we respectively selected crRNA-lef1 (S/N= 100.00) and crRNA-capB3 (S/N= 50.00) as the best *lef* and *capB* crRNAs for subsequent detection purposes (Figure 1A). An “exhaustive” method was used for the crRNA selection of SNP sites in *plcR* and CR5. Six crRNAs in which the PAM sequence and the SNP site were generally separated by 0–5 bases were designed to target the coding strand. Six corresponding crRNAs were also designed to target the non-coding strand (Figure 1B). Twelve possible crRNAs were screened and tested in three independent replicates. According to the results, the best crRNAs for *plcR* and CR5 were crRNA-pR6 (S/N= 3.68) and crRNA-CR3 (S/N= 14.93), respectively, which were therefore selected for subsequent detection experiments (Figure 1C). The selected crRNAs and their detection target sequences are listed in Table 1.

**Table 1.** Optimized CRISPR-derived RNAs for target sequences.

Target site	Target sequences (5′−3′)	PAM sequence	Primers (5′−3′)		crRNA^#^ (5′−3′)
lef	TTTCTTACATCAAGATTAATT	TTTC	lef-F	AAAGCATCAATATTTTGAATATCCCTTTTATACTGC	AAUUUCUACUGUUGUAGAUUUACAUCAAGAUUAAUU
			lef-R	CTTGATATTCAACCATATGATATTAATCAAAGGTTGC	
capB	TTTCCTCATCAATCCCAAGAG	TTTC	capB-F	GCATTCAACATACCACGGAATGCTG	AAUUUCUACUGUUGUAGAUCUCAUCAAUCCCAAGAG
			capB-R	CATGGTCTTCCCAGATAATGCATCG	
plcR	AAAGCGCTTATTCGTATTGAT	TTTG*	plcR-F	AGCTTTATTTGCATGAC**TTTG**CGCTT	AAUUUCUACUGUUGUAGAUCGCUUAUUCGUAUUGAU
			plcR-R	GAGTTTGATGTGAAGGTGAGACATAATCATGC	
CR5	AAGTAACACAGAAGATAAGTC	TTTT*	CR5-F	CAACGAGCACGACACCAGA**TTTT**AA	AAUUUCUACUGUUGUAGAUAACACAGAAGAUAAGUC
			CR5-R	ATCAGCTCTCCTAGTGAGAACTGATT	

*Introduction of PAM sequences by PCR or RPA primers for SNP site detection.

#The crRNA consists of a 19-nt universal sequence (AAUUUCUACUGUUGUAGAU) located 17-nt downstream of the PAM sequence.

The selected crRNAs were used in the Cas12a detection system in combination with RPA. Genomic nucleic acid (15 aM/reaction) was subjected to RPA, and subsequent Cas12a detection was able to fully distinguish *B. anthracis* from *B. cereus*. Three independent repeat experiments were conducted per detection reaction. All *B. anthracis* detection targets emitted strong fluorescence signals, whereas those of *B. cereus* (the negative control) emitted almost no fluorescence (Figure 1D). These results, which are consistent with those obtained using fluorescent probes and LFD strips (Figure 1E), were preliminary confirmation that the selected crRNAs had good specificities.

### A new visualization system based on the G-quadruplex-hemin DNAzyme

**Figure 2.**
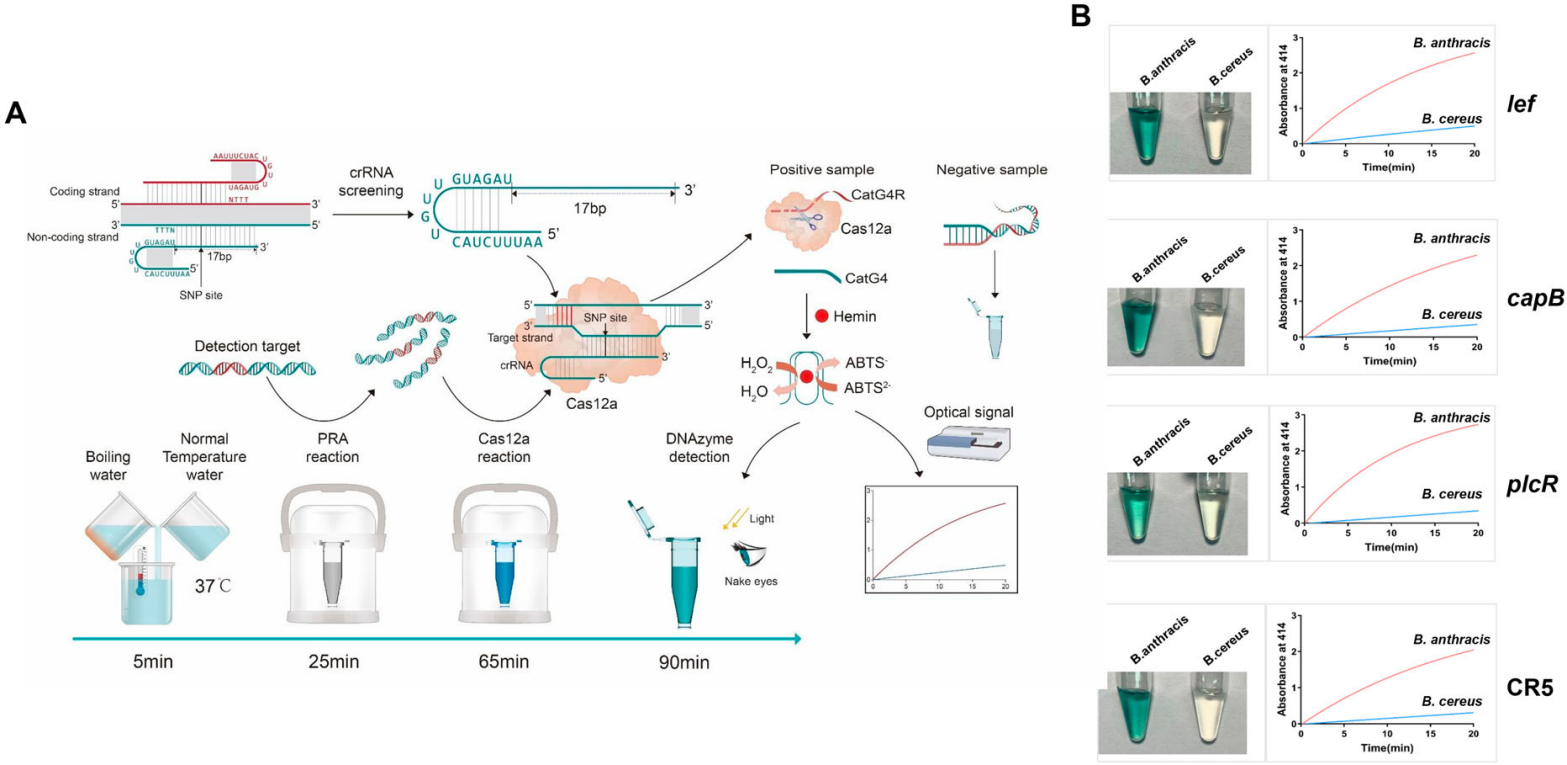
**The visualization system based on the peroxidase activity of DNAzyme**. **(A)** Schematic diagram demonstrating the principle of the DNAzyme visualization system. Boiling water and room-temperature water are mixed to achieve a temperature of 37°C as measured with a thermometer. RPA and Cas12a reactions are consecutively performed in thermos cups. CatG4R antisense DNA is added as a detection probe for the Cas12a reaction. After the cleavage reaction of CatG4R by Cas12a, CatG4 nucleic acid is then added. CatG4 and hemin can form an activated G-quadruplex-hemin complex, which catalyses ABST^2-^ and H_2_O_2_ to produce ABST^-^ and turn the solution green. If Cas12a cannot cleave the CatG4R, CatG4 complementarily pairs with its antisense molecule CatG4R to form double-stranded DNA; because CatG4 cannot exert its enzyme activity, the solution remains colourless. The entire detection process can be completed within 90 min without electrical power. (**B)** Colour changes in the four targets. The genomic DNAs in the *B. anthracis* samples all generated green solutions, whereas those from *B. cereus* remained colourless. The results of absorbance measurements at 414 nm are consistent with the visual observations.

DNAzymes are catalytic nucleic acids, among which, G-quadruplex-hemin has peroxidase activity. We combined G-quadruplex-hemin and the Cas12a detection system to develop a new detection method that gives results visible to the unaided eye. The basis of this system is as follows: after cleavage of the antisense molecules by Cas12a, CatG4 and hemin can form an activated G-quadruplex-hemin complex whereby ABST^2-^ and hydrogen peroxide are catalysed to ABST^-^, thereby turning the solution green. When Cas12a cannot cleave the antisense probe, however, CatG4 complementarily pairs with the CatG4R antisense molecule to form double-stranded DNA and an inactive enzyme; in this case, the solution remains colourless. The whole detection process can be completed within 90 min without electrical power, with the correct temperature conditions achieved with boiling water and thermos cups rather than electrical equipment (Figure 2A).

Identification of the four *B. anthracis* targets was further confirmed using G-quadruplex-hemin (Figure 2B). In the presence of this DNAzyme, all solutions containing *B. anthracis* genomic DNA turned green, whereas the *B. cereus* samples (1.5 × 10^3^ aM/reaction) remained colourless. The results of absorbance measurements at 414 nm were consistent with these visual observations.

### Specificity and sensitivity of fluorescence and DNAzyme reporters based on RPA–Cas12a detection

**Figure 3.**
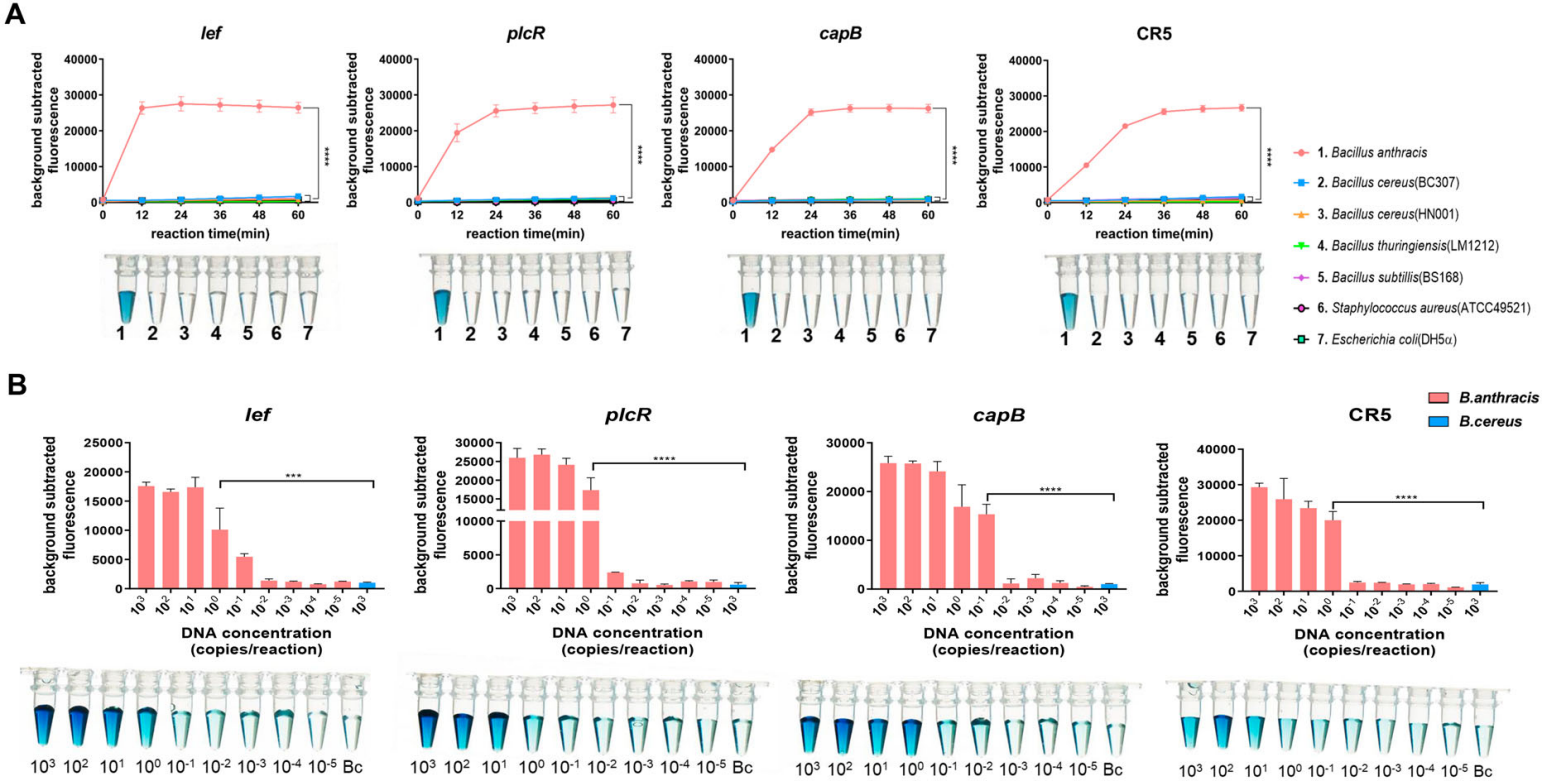
**Specificity and sensitivity of the RPA–Cas12a detection method**. **(A)** RPA-Cas12a detection specificity (three replicates; bars represent the mean ± SD). *B. anthracis* and non-anthracis strains showed statistically significant differences (two-way repeated measures ANOVA, *p* < 0.0001). In the DNAzyme system, solutions of all *B. anthracis* genomic DNA samples turned green, whereas samples of the other strains remained colourless. (**B)** Histograms showing RFU values at 36 min into the test (three technical repetitions otherwise noted). When the concentration reached 1 copy/reaction. RFUs of *plcR* (8 out of 8 replicates amplified), *lef* (6 out of 8 replicates amplified) and CR5 (5 out of 8 replicates amplified) exhibited significant statistical differences compared with the negative control (eight technical repetitions, *t*-test, *p* < 0.001). RFU values *capB* (5 out of 8 replicates amplified), were still significantly different than those of the negative control when the concentration reached 10^−1^ copies/reaction (*t*-test, *p* < 0.001). The LOD of DNAzyme reporter reached 1 copy/reaction for plasmid targets (*lef* and *capB*), and 10 copies/reaction for chromosomal SNP targets (*plcR* and CR5).

To further verify the specificity of the RPA–Cas12a detection method, Genomic nucleic acid (about 15 aM/reaction) of six strains other than *B. anthracis* were detected. *Bacillus cereus* group isolates, including *B. cereus* BC307, *B. cereus* HN001, and *B. thuringiensis* LM1212, were selected as genetically similar reference strains. In addition, *B. subtilis* str. 168, a model *Bacillus* strain, was chosen as a genetically related but distinguishable strain. Finally, gram-positive *S. aureus* ATCC49521 and gram-negative *E. coli* DH5α were selected as genetically distant strains. Fluorescence probe-based detection exhibited good specificity, with *B. anthracis* displaying strong fluorescence signals for all four targets and the other species emitting almost no fluorescence (Figure 3A). The DNAzyme reporting system was characterized by good specificity as well: solutions of all *B. anthracis* genomic DNA samples turned green, whereas samples of the other strains remained colourless.

Genomic DNA from *B. anthracis* was 10-fold serially diluted to test the sensitivity of the RPA–Cas12a detection method. The DNA concentration in each reaction mixture was 1.5 × 10^3^–1.5 × 10^−5^ aM, which corresponds to approximately 10^3^–10^−5^ genome copies. The time to reach the highest fluorescence value was 12–36 min (Figure 3A). Therefore, 36 min was set as the reaction detection time in subsequent experiments. A histogram of fluorescence values taken 36 min into the reaction is shown in Figure 3B. When the concentration reached 10^−1^ copies/reaction, the fluorescence values for *capB* (5 out of 8 replicates successfully amplified) still differed significantly from that of the negative control, which was performed with 10^3^ *B. cereus* genome copies (*t*-test, *p* < 0.0001). The sensitivity of detection of *plcR* (8 out of 8 replicates successfully amplified), *lef* (6 out of 8 replicates successfully amplified) and CR5(5 out of 8 replicates successfully amplified) targets thus reached 1 copy/reaction. In summary, 1 copy per reaction is conservatively estimated to be the sensitivity level for the entire RPA-Cas12a detection system.

The DNAzyme reporter also exhibited high sensitivity (Figure 3B), with the LOD of plasmid targets (*lef* and *capB*) reaching 1 copy/reaction. In contrast, the sensitivity level for chromosomal SNP targets (*plcR* and CR5) was only 10 copies/reaction, however, the level of this sensitivity is sufficient for POCT.

We also performed qPCR based on TaqMan probes for comparison with the CRISPR-Cas12a method. The qPCR results are shown in Table 2, and the primers used and the relevant amplification curve are displayed in Supplementary files Table S5 and Figure S3, respectively. The LOD of plasmid targets (*lef* and *capB*) and chromosomal SNP targets of *plcR* was 1 copy/reaction, while the LOD of the CR5 target was 10 copies/reaction.

**Table 2.** Average Cycle Threshold Values for qPCR Sensitivity Test^a^

Copies/μL	Average Ct			
	capB	lef	CR5	plcR
10^3^	24.7±0.2	23.8±0.4	24.4±0.7	26.4±0.1
10^2^	29.7±0.1	28.5±0.2	29.3±0.5	31.5±0.0
10^1^	34.1±0.9	35.0±0.4	34.1+0.8	36.80±0.5
10^0^	38.7±0.7^b^	37.2±1.1	NA	39.25±0.1^c^
10^–1 d^	40.5^e^	38.6±3.5^f^	NA	NA
LOD	1 copies/μL	1 copies/μL	10 copies/μL	1 copies/μL

NA. no amplification

^a^ Cycle threshold (Ct) values are an average of 4 repetitions unless otherwise noted.

^b^ Of the 4 replicates, 3 amplified

^c^ Of the 4 replicates, 3 amplified

^d^ All samples of 10^–1^ copies were replicated 10 times, the Cts from samples that amplified were averaged.

^e^ Of the 10 replicates, 1 amplified.

^f^ Of the 10 replicates, 2 amplified.

### Detection of *B. anthracis* vegetative cells and spores using the CRISPR–Cas12a system

For this experiment, *B. anthracis* culture (vegetative cells) and purified spores were centrifuged and then resuspended in 1 mL double distilled water. The resuspended culture and spores were 10-fold serially diluted separately, and the viable bacteria were counted. After heating the 10^7^–10^0^ CFU/mL dilutions in a 100°C water bath, 1 µL of each solution was used as a template for RPA.

**Figure 4.**
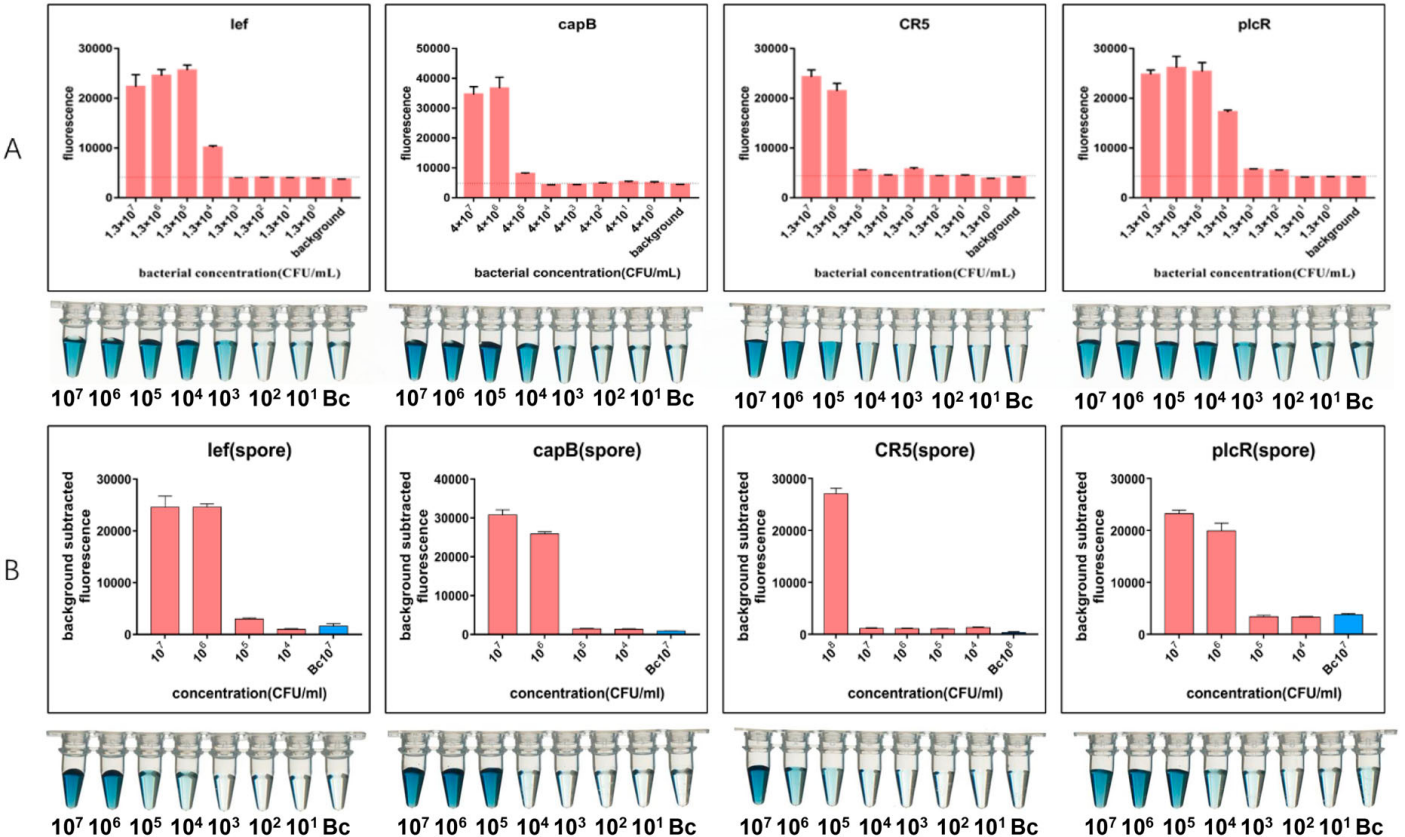
**Detecting B. anthracis vegetative cells and spores with the CRISPR-Cas12a system**. **(A)** Detection results for vegetative cells. In the fluorescence reporting system, LODs were 10^4^ CFU/mL for *lef* and *plcR* targets, 10^6^ CFU/mL for *capB* and CR5 targets. In DNAzyme reporting system, LODs were 10^3^ CFU/mL for *lef* and *plcR* targets, 10^4^ CFU/mL for *capB* and 10^5^ CFU/mL for CR5 targets (**B)** Detection results for spores. In the fluorescence reporting system, LODs were 10^6^ CFU/mL for *lef*, *plcR* and *capB* targets and 10^8^ CFU/mL for CR5 target. In DNAzyme reporting system, LODs were 10^6^ CFU/mL for *lef,* targets, 10^5^ CFU/mL for *plcR* and *capB* targets, 10^7^ CFU/mL for CR5 targets.

When the fluorescence reporting system was applied to vegetative cells (Figure 4A), the LOD of *lef* and *plcR* targets was 10^4^ CFU/mL (∼10 copies/reaction). The sensitivity of the DNAzyme reporter was better: the LOD of *lef* and *plcR* targets was 10^3^ CFU/mL (∼1 copy/reaction). Although the colour response of the 10^3^ CFU/mL sample was weaker than that of the more concentrated solution, positive and negative results could still be distinguished by visual inspection. The LOD for *capB* and CR5 targets using the fluorescence reporting system was 10^6^ CFU/mL (∼10^3^ copies/reaction), whereas that obtained with the DNAzyme reporter was 10^4^ CFU/mL (∼10 copies/reaction) for *capB* and 10^5^ CFU/mL (∼10^2^ copies/reaction) for the CR5 target.

Regarding the detection of *B. anthracis* spores using the fluorescence reporting system, we obtained LODs of *lef, plcR* and *capB* targets of 10^6^ CFU/mL (∼10^3^ copies/reaction), whereas that for the CR5 target was 10^8^ CFU/mL (∼10^5^ copies/reaction). Similar to the results obtained with vegetative cells, the sensitivity of the DNAzyme reporter when applied to spores was better than that of the fluorescence reporting system. Although the LOD of the *lef* target was similar between the two reporting systems, the LODs of the other three targets were 10 times lower when the DNAzyme reporter was used (Figure 4B).

The results of *B. anthracis* detection using vegetative cells and spores were quite different from those of genomic DNA samples. This discrepancy may be due to the different capabilities of RPA between complex and simple samples. The higher sensitivity of the DNAzyme reporter maybe because the enzyme-catalysed reaction has a signal-amplifying effect relative to the level of released fluorescence signals.

## Discussion

With the advancement of molecular detection technology, an increasing number of detection methods are now being applied to identify *B. anthracis*, with next-generation sequencing (NGS) becoming the gold standard. NGS is not suitable for use by most disease control agencies, however, especially given that the areas where anthrax is present are often very remote. Furthermore, a small number of *B. cereus* sequences have been erroneously deposited in the GenBank database as *B. anthracis* even though their pXO1 and pXO2 plasmids are missing (e.g. GenBank assembly accession nos. GCA_003410355.1 and GCA_003410255.1). Although these accessions were sequenced by laboratories specializing in microbiological research, the wrong conclusions can still be drawn during analyses. Resolving this issue is obviously not practical during *B. anthracis* field detection. A detection method that is easy to implement with readily interpretable results has thus been needed.

In this study, we developed a rapid detection method based on the CRISPR-Cas12a system that does not require access to electricity. Using this system, we selected two target plasmid genes (*lef* and *capB*) to screen samples for pathogenic *B. anthracis*. We concurrently chose two chromosomal SNP targets (*plcR* and CR5) to detect *B. anthracis* DNA in samples. Notably, a completely new SNP site in the CR5 target was selected from the highly conserved *B. anthracis* CRISPR sites predicted in the 1,992 GenBank strains used to verify the SNP site, namely, 252 *B. anthracis*, 1,118 *B. cereus*, and 622 *B. thuringiensis* strains (Supplementary file, Figure S1). This total encompasses all relevant strains in the GenBank database as of September 2020. The entire detection process can be completed within 90 min without electrical power. Instead of electrical equipment, the only requirements to reach the correct temperature conditions are boiling water and thermos cups (Figure 2A). The fact that the test results are visible to the unaided eye will help reduce the cost of professional training for the operators.

Since its invention, the CRISPR-Cas-based detection system has attracted the attention of researchers worldwide, and a large number of strategies have been developed. Because the trans-cleavage activity of the Cas protein is relatively insufficient, most CRISPR-Cas-derived techniques must include pre-amplification. Intending to ensure sensitivity, future CRISPR-Cas-based detection development needs to eliminate this amplification requirement. Current research has generally focused on increasing the target concentration, coupling electrochemical sensors to collect weak signals, and amplifying reported signals. SELEX (Systematic Evolution of Ligands by Exponential Enrichment) technology has been used to develop many DNAzymes, RNAzymes, and aptamers that have been widely applied in various detection technologies and molecular biosensors. In essence, DNAzyme is a short single-stranded DNA molecule whose catalytic activity appears to be easily introduced into a detection system via the trans-cleavage activity of Cas12a. When DNAzyme CatG4 was used as a probe in our study, however, solutions containing either *B. anthracis* or *B. cereus* turned green, which means the two strains were not distinguishable using this detection approach (Supplementary file, Figure S2A). A possible explanation for this outcome is that the three guanylic acids in series (-GGG-) remained in the solution after cleavage of the CatG4 probe by Cas12a and formed G4 complexes that still had peroxidase activity [27]. Another problem with using the CatG4 sense strand as a probe is that buffer components such as Tris-HCl can also interfere with the catalytic reaction of DNAzyme, causing false-positive results (Supplementary file, Figure S2B). We, therefore, used the complementary sequence CatG4R as the detection probe, which caused the enzymatic activity of DNAzyme to be blocked by complementary base pairing. Compared with the direct colour development method using CatG4, this approach showed stronger resistance to interference, as a change in the colour of the solution to green could be judged as a positive reaction, and the false positive rate would also be reduced. Additionally, a complementary CatG4R sequence could be designed not only as a DNA molecule but also as an RNA molecule (Supplementary file, Figure S2C). This modification makes the DNAzyme-based visualization solution compatible with other detection systems, such as the Cas13a-based SHERLOCK.

We also tested vegetative cells and spores using the CRISPR–Cas12a system. Enrichment of vegetative cells from clinical samples or spores from environmental samples is, however, a common challenge faced by all known detection methods. Regardless of the detection assay and the nucleic acid extraction method, raw samples must be processed efficiently [28]. Many methods have been reported for the recovery of *B. anthracis*, including non-specific enrichment with carrier media and centrifugation, specific immunomagnetic enrichment, phage coated magnetoelastic enrichment, peptide aptamers, and DNA aptamers. Therefore, many methods can be used for sample pre-processing for the CRISPR-Cas12a system described herein. Fortunately, most magnetic-based methods do not require electrical power.

## Conclusions

We developed a complete detection system for *B. anthracis* based on Cas12a. The entire detection process can be completed within 90 min without electrical power and with single-copy level sensitivity. We also developed an unaided-eye-visible biosensing system based on G4-DNAzyme for use with our CRISPR-Cas12a detection system. Our newly developed set of CRISPR-Cas12a *B. anthracis* detection assays can be performed without the need for electricity, and the incorporation of a visualization solution based on DNAzymes broadens its applicability.

## Declaration of competing interests

All authors have approved the manuscript and the authors have no conflicts of interest to declare.

## Supplementary Material

Supplemental Material
