# Supplementary material for: A CRISPR/Cas12a-based DNAzyme visualization system for rapid, non-electrically dependent detection of Bacillus anthracis
Source: Emerg Microbes Infect. 2022 Feb 1;11(1):428–37. doi: 10.1080/22221751.2021.2012091 (PMC8812752; doi:10.1080/22221751.2021.2012091)
Supplement: Supplemental Material [file TEMI_A_2012091_SM4482.docx]

Supplementary File

**contents**

[**Figure S1 Specific SNP sites in the CR5 target** 2](#_Toc85769548)

[**Figure S2 Design and expansion of the DNAzyme color reaction** 3](#_Toc85769549)

[**Figure S3 qPCR detection of lef, capB, CR5 and plcR sites** 4](#_Toc85769550)

[**Table S1 Genomic DNA concentration and copy number** 5](#_Toc85769551)

[**FiguresS4. Detection of ciprofloxacin resistance in *B. anthracis*** 6](#_Toc85769552)

[**Table S2 PCR amplification primers and crRNAs for the screened target sequences** 8](#_Toc85769553)

[**Table S3 CRISPR-Cas12a reaction system constituents (20 μL**) 10](#_Toc85769554)

[**Table S4 RPA amplification primers and reaction system** 11](#_Toc85769555)

[**Table S5 qPCR amplification primers and TaqMan probe** 11](#_Toc85769556)


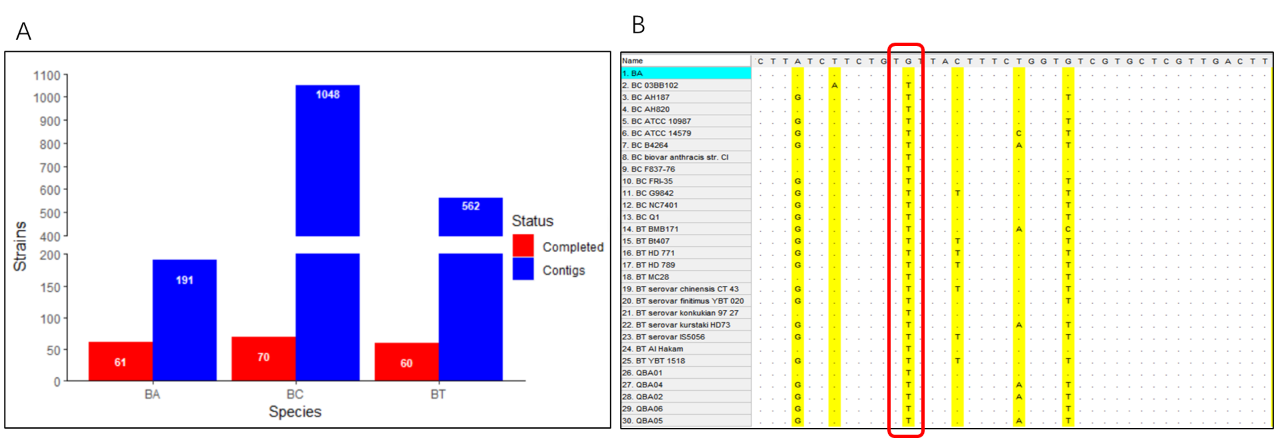


**Figure S1 Specific SNP sites in the CR5 target**

A: GenBank strains (n=1992) were used to verify the SNP site in the CR5 target; they included 252 *B. anthracis* strains, 1118 *B. cereus* strains, and 622 *B. thuringiensis* strains. This covers all relevant strains in the GenBank database (September 2020). Strains with completed sequences are shown in red. Sequences remaining as contigs are marked in blue. B: A part of the sequence alignment results is displayed. A specific SNP site is marked by a red rectangle in the CR5 target. This nucleotide is ‘G’ in *B. anthracis* and ‘T’ in *B. cereus* and *B. thuringiensis*.

**Design and expansion of the DNAzyme colour reaction**

**Methods: A.** ProbeCatG4: The Cas12a reaction was performed according to the method in Table S3, where the CatG4 (whose sequence is 5′-TGGGTAGGGCGGGTTGGGAAA-3′) was added to the Cas12a detection system as the reporter probe. 2 μL of hemin (100 μmol/L) and 72 μL of MES buffer (0.1 mol/L, pH 4.7) were added after Cas12a reaction. The thoroughly mixed solution contained 2 μL of ABTS (50 mmol/L), and 2 μL of H_2_O_2_ (3% v/v) ProbeCatG4: Reaction was performed according to the method in section 2.5.

B. The reaction contained nuclease-free water 20μ, CatG4 probe (10 μM) 2μL, 2μL of hemin (100 μmol/L), 2μL of ABTS (50 mmol/L), and 2μL of H_2_O_2_ (3% v/v). Cas12a nuclease (0.15mg/mL) 1μL. As the 72uL reaction buffer, nuclease-free water, NEB buffer 3.0, nuclease-free water contained 1uL LbCas12a nuclease (0.15mg/mL), nuclease-free water contained 1uL 1uL crRNA (10mmol/L) and nuclease-free water contained 20uL PRA reaction product were used separately.

C. Reaction was performed according to the method in section 2.5. The difference was to use CatG4R-RNA (whose single-stranded RNA sequence is 5′-UUUCCCAACCCGCCCUACCCA-3′) instead of CatG4R.


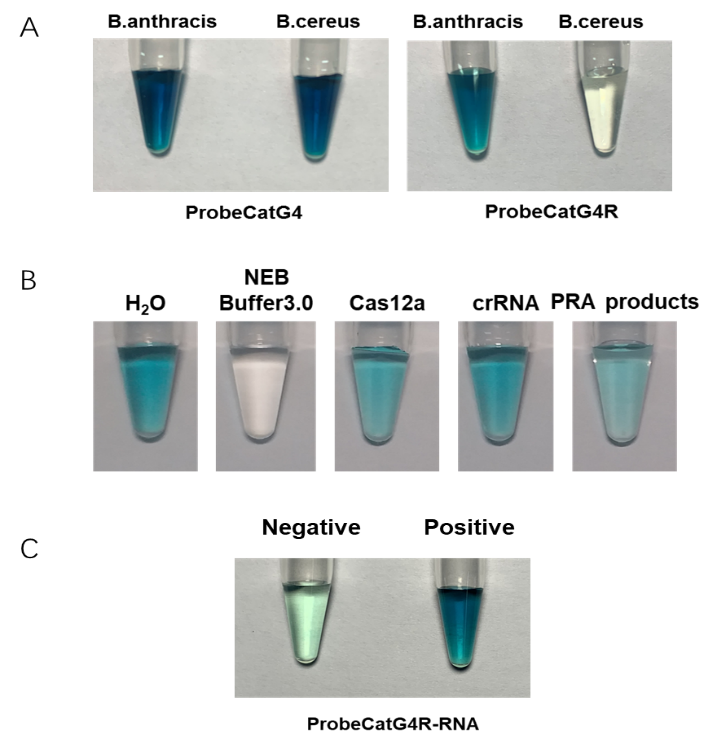


**Figure S2 Design and expansion of the DNAzyme colour reaction**

A: The detection results for *B. anthracis* and *B. cereus* both showed green colour when CatG4 was used as the probe. The *B. anthracis* samples produced green-coloured solutions, whereas the *B. cereus* samples remained colourless when antisense CatG4R was used as the probe. B: Effects of the CRISPR-Cas12a reaction system components on the peroxidase activity of DNAzyme.

Colour development by DNAzyme was blocked by NEB Buffer 3.0, which contains Tris-HCl. C: Antisense RNA CatG4R-RNA was used as a probe instead of DNA. When antisense RNA was cleaved, the solution turned green, which represents a positive result. When the antisense RNA could not be cut, the solution turned very light green, which represents a negative result.

**qPCR detection**

The qPCR based on TaqMan probes were performed for comparison with CRISPR-Cas12a methods. Real-time PCRs were conducted in 10-μL reaction mixtures that contained 600 nM of both forward and reverse primers, 250nM of each probe (Table S5), 10 μL Luna Universal Probe qPCR Master Mix (New England Biolabs, Ipswich, MA, USA), and 1μL of the template. Thermal cycling was performed on a Bio-Rad CFX96 Connect system under the following conditions: 95°C for 2 min, and 40 cycles of 95°C for 15 s and 60°C for 1 min (with plate read).

**
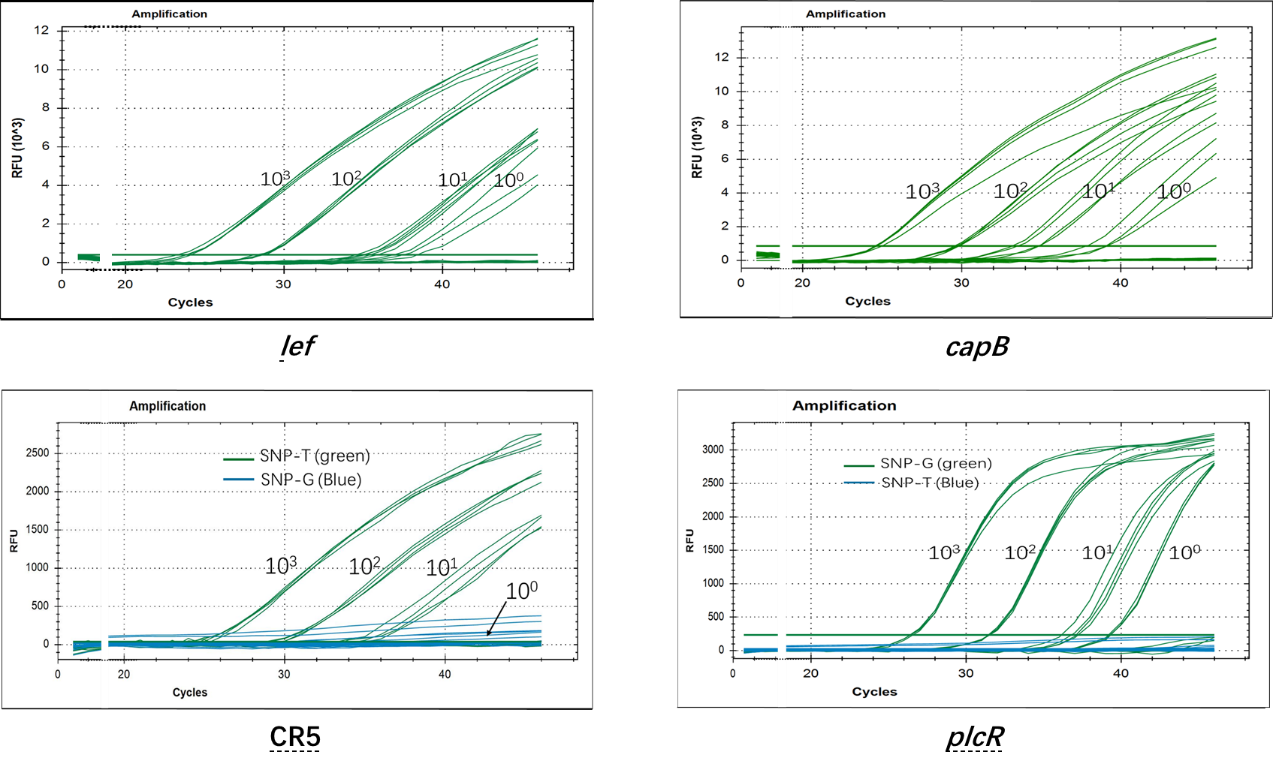
**

**Figure S3 qPCR detection of lef, capB, CR5 and plcR sites**

Results of triplicate analysis of 10-fold serial dilutions of DNA from *B. anthracis* strains (shown in green). The average cycle threshold values across the four replicates were shown in Table 2. DNA from *B. cereus* strain BC307 was set as the negative controls (shown in blue).

**Genomic DNA concentration and copy number**

The quantitation of genomic DNA was performed with iQuant™ NGS-BR dsDNA Assay Kit using Qubit 3.0 Fluorometer (Thermo Fisher Scientific Inc., Waltham, MA, USA). DNA copies number were determined following formula (6.02 × 10^23^) × (ng/μL ×10^−9^)/ (DNA length × 660) = copies/μL. The Digital PCR were entrusted to Sangon Biotech (Shanghai, China). The results are shown in Table S1.

**Table S1 Genomic DNA concentration and copy number**

| Target | Sample | Concentration (ng/μL) | digital PCR measured Copy number (μL^-1^) | Estimated copy number (μL^-1^) |
| --- | --- | --- | --- | --- |
| *plcR* | A16PI2 | 52 ng/μL | 1713333 | 8980627 |
| CR5 |  |  | 1886667 |  |
| *lef* |  |  | 8060000 |  |
| *capB* | A16Q1 | 73 ng/μL | 4940000 | 12773390 |

**Detection of ciprofloxacin resistance in *B. anthracis***

*Bacillus anthracis* is relatively conserved genetically, but some mutations in this pathogen can lead to antibiotic resistance[1-3]. Two common mutations, both of which confer ciprofloxacin resistance, were selected for drug-resistance detection: C254→T in gyrA (DNA gyrase) and C242→T in parC (type IV DNA topoisomerase).

The gyrA (C254→T) and parC (C242→T) open reading frames were cloned into pUC19 to simulate quinolone-resistant strains containing these mutations (final concentration = one copy per reaction). crRNA screening was performed as described above, and crRNA-gR2 and crRNA-p5 were selected for use in subsequent Cas12a-based detection in combination with RPA (Figure 5A). Using this detection strategy, we were able to clearly distinguish *B. anthracis* strain A16PI2, which lacked the resistance mutations, from the plasmids containing the gyrA and parC resistance mutations (Figure 5B). The visual images shown in Figure 5C are consistent with measured fluorescence values, both of which were positive for the detection of the plasmid-located resistance mutations and negative for B. anthracis A16PI2.


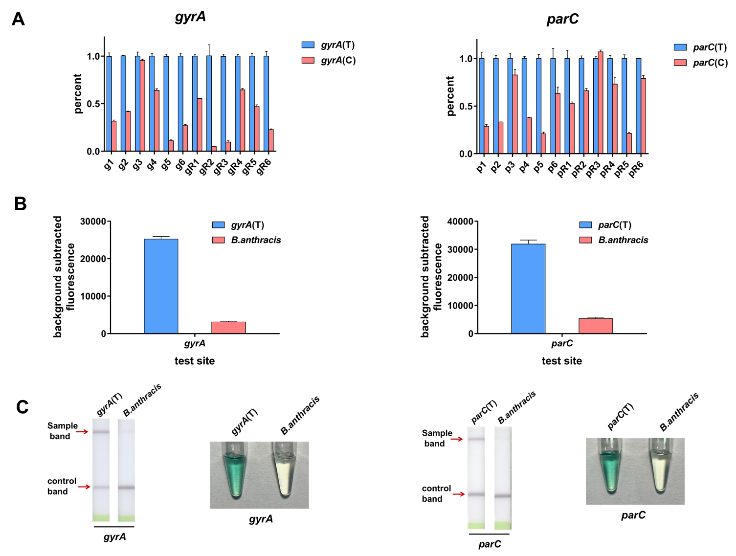


**FiguresS4. Detection of ciprofloxacin resistance in B. anthracis using the CRISPR/Cas12a-based DNAzyme visualization system**

(A) Screening and optimization of crRNAs for gyrA and parC gene targeting. All 12 possible crRNAs were screened for SNP detection. Histograms were used to show the difference between the positive and negative results, and crRNA-gR2 (for gyrA) and crRNA-p5 (for parC) were selected as the best crRNAs for subsequent detection. (B) Differentiation of the sensitive genotype from the quinolone-resistant genotype using RPA–Cas12a detection. The gyrA (C254→T) and parC (C242→T) open reading frames were cloned into pUC19 [53] to simulate quinolone resistance in the strains containing these mutations. Plasmids containing the resistance mutations generated strong fluorescence signals, whereas B. anthracis strain A16PI2, which lacks these mutations, generated much weaker fluorescence signals. The sensitive and resistant genotypes were readily distinguishable.

1. Easterday WR, Ert MNV, Simonson TS, et al. Use of Single Nucleotide Polymorphisms in the plcR Gene for Specific Identification of Bacillus anthracis. Journal of Clinical Microbiology. 2005;43(4):1995-1997.

2. Rafał G, Zasada AA, Noura R, et al. Specific Bacillus anthracis identification by a plcR-targeted restriction site insertion-PCR (RSI-PCR) assay. FEMS Microbiology Letters. (1):1.

3. Wang D, Wang B, Zhu L, et al. Genotyping and population diversity of Bacillus anthracis in China based on MLVA and canSNP analysis. Microbiological Research. 2020;233:126414.

**Table S2 PCR amplification primers and crRNAs for the screened target sequences**

| Target site | Target sequences  5’-3’ | PAM sequence | Primers  5’-3’ | | crRNA^#^  5’-3’ |
| --- | --- | --- | --- | --- | --- |
| lef-1 | TTTCTTACATCAAGATTAATT | TTTC | lef-1F | AAAGCATCAATATTTTGAATATCCCTTTTATACTGC | AAUUUCUACUGUUGUAGAU UUACAUCAAGAUUAAUU |
|  |  |  | lef-1R | CTTGATATTCAACCATATGATATTAATCAAAGGTTGC |  |
| lef-2 | TTTCGCAAATACTTCTTGTAC | TTTC | lef-2F | CGATGCTGTGGCTCGATATAATATGCA | AAUUUCUACUGUUGUAGAUGCAAAUACUUCUUGUAC |
|  |  |  | lef-2R | GCATCTGATTCAGATGGACAAGATCTTTTATT |  |
| lef-3 | TTTCAAACGCTCATTATCTAA | TTTC | lef-3F | CTCGAGTATCTGGTGATAATTGGATTCTCC | AAUUUCUACUGUUGUAGAUAAACGCUCAUUAUCUAA |
|  |  |  | lef-3R | CTAGGTGCGGATTTAGTTGATTCCACT |  |
| lef-4 | TTTCATACAAATAAATTTTAT | TTTC | lef-4F | AGTGGAATCAACTAAATCCGCACCTAG | AAUUUCUACUGUUGUAGAUAUACAAAUAAAUUUUAU |
|  |  |  | lef-4R | GATGCTTTATTACATCAATCCATTGGAAGTACC |  |
| lef-5 | TTTCCATTCATTCAATATTAA | TTTC | lef-5F | ATCGGTAAAAACAAATCTTCCATTACCATCAAC | AAUUUCUACUGUUGUAGAUCAUUCAUUCAAUAUUAA |
|  |  |  | lef-5R | CAACGTGCATAATAGATATGCATCCAATATTG |  |
| capB-1 | TTTCTGATGGCAACGTTGTTC | TTTC | capB-1F | ATGTTTACTCGAATTGGGATAGAATTGAGCCT | AAUUUCUACUGUUGUAGAU UGAUGGCAACGUUGUUC |
|  |  |  | capB-1R | AGCAGGAAGGGATTAATACAGATGATCTTCATA |  |
| capB-2 | TTTCAAAAGCACTTGTAATAG | TTTC | capB-2F | AGTTCCAATACTCTTGCGTTGGAATATCTCCTT | AAUUUCUACUGUUGUAGAUAAAAGCACUUGUAAUAG |
|  |  |  | capB-2R | TGACCGCGTTGATCGTACTGAGCAGTTTGCTAG |  |
| capB-3 | TTTCCTCATCAATCCCAAGAG | TTTC | capB-3F | GCATTCAACATACCACGGAATGCTG | AAUUUCUACUGUUGUAGAUCUCAUCAAUCCCAAGAG |
|  |  |  | capB-3R | CATGGTCTTCCCAGATAATGCATCG |  |
| capB-4 | TTTCCCTACAGTCTTATATTT | TTTC | capB-4F | CCAATATATCATTCGCGCAGATGTACCAGTTGT | AAUUUCUACUGUUGUAGAUCCUACAGUCUUAUAUUU |
|  |  |  | capB-4R | GGAATTCGAGGTAAATCTACCGTTACAAGACTA |  |
| capB-5 | TTTCGACTTAAGTAGAGTCGT | TTTC | capB-5F | CTGATGGCAACGTTGTTCCCATATACCATAAAT | AAUUUCUACUGUUGUAGAUGACUUAAGUAGAGUCGU |
|  |  |  | capB-5R | GATGAATTCGGTGTTGTATATGCAGGGAACCAC |  |
| CR5-1 | CTGTGTTACTTTCTGGTGTCG | TTTT | CR5-1F | CTTGTTGATTTCTCTTCTTTTTGAGACTTATCTT**TTTT** | AAUUUCUACUGUUGUAGAUGTTACTTTCTGGTGTCG |
|  |  |  | CR5-R | CAAAGGCAACGAATGCCTCTGG |  |
| CR5-2 | TCTGTGTTACTTTCTGGTGTC | TTTG | CR5-2F | CTTGTTGATTTCTCTTCTTTTTGAGACTTATCT**TTTG**T | AAUUUCUACUGUUGUAGAUTGTTACTTTCTGGTGTC |
|  |  |  | CR5-R | Ditto |  |
| CR5-3 | TTCTGTGTTACTTTCTGGTGT | TTTT | CR5-3F | CTTGTTGATTTCTCTTCTTTTTGAGACTTATC**TTTT**GT | AAUUUCUACUGUUGUAGAUGTGTTACTTTCTGGTGT |
|  |  |  | CR5-R | Ditto |  |
| CR5-4 | CTTCTGTGTTACTTTCTGGTG | TTTC | CR5-4F | CTTGTTGATTTCTCTTCTTTTTGAGACTTAT**TTTC**TGT | AAUUUCUACUGUUGUAGAUTGTGTTACTTTCTGGTG |
|  |  |  | CR5-R | Ditto |  |
| CR5-5 | TCTTCTGTGTTACTTTCTGGT | TTTT | CR5-5F | CTTGTTGATTTCTCTTCTTTTTGAGACTTA**TTTT**CTGT | AAUUUCUACUGUUGUAGAUCUGUGUUACUUUCUGGU |
|  |  |  | CR5-R | Ditto |  |
| CR5-6 | ATCTTCTGTGTTACTTTCTGG | TTTT | CR5-6F | CTTGTTGATTTCTCTTCTTTTTGAGACTT**TTTT**TCTGT | AAUUUCUACUGUUGUAGAUTCTGTGTTACTTTCTGG |
|  |  |  | CR5-R | Ditto |  |
| CR5-R1 | GTAACACAGAAGATAAGTCTC | TTTA | CR5-R1F | CAACGAGCACGACACCAGAAA**TTTA** | AAUUUCUACUGUUGUAGAUCACAGAAGATAAGTCTC |
|  |  |  | CR5-RR | ATCAGCTCTCCTAGTGAGAACTGATT |  |
| CR5-R2 | AGTAACACAGAAGATAAGTCT | TTTA | CR5-R2F | CAACGAGCACGACACCAGAA**TTTA**A | AAUUUCUACUGUUGUAGAUACACAGAAGATAAGTCT |
|  |  |  | CR5-RR | Ditto |  |
| CR5-R3 | AAGTAACACAGAAGATAAGTC | TTTT | CR5-R3F | CAACGAGCACGACACCAGA**TTTT**AA | AAUUUCUACUGUUGUAGAUAACACAGAAGATAAGTC |
|  |  |  | CR5-RR | Ditto |  |
| CR5-R4 | AAAGTAACACAGAAGATAAGT | TTTG | CR5-R4F | CAACGAGCACGACACCAG**TTTG**TAA | AAUUUCUACUGUUGUAGAUTAACACAGAAGATAAGT |
|  |  |  | CR5-RR | Ditto |  |
| CR5-R5 | GAAAGTAACACAGAAGATAAG | TTTA | CR5-R5F | CAACGAGCACGACACCA**TTTA**GTAA | AAUUUCUACUGUUGUAGAUGTAACACAGAAGATAAG |
|  |  |  | CR5-RR | Ditto |  |
| CR5-R6 | AGAAAGTAACACAGAAGATAA | TTTA | CR5-R6F | CAACGAGCACGACACC**TTTA**AGTAA | AAUUUCUACUGUUGUAGAUAGTAACACAGAAGATAA |
|  |  |  | CR5-RR | Ditto |  |
| plcR-1 | CGAATAAGCGCTTTGTCATGC | TTTA | plcR-1F | ATGCAAAAGCATTATACTTGGACAATCAATA**TTTA** | AAUUUCUACUGUUGUAGAUUAAGCGCUUUGUCAUGC |
|  |  |  | plcR-R | CTAGGCATTCAACTTTTCGATAGTATAACTGTCC |  |
| plcR-2 | ACGAATAAGCGCTTTGTCATG | TTTA | plcR-2F | ATGCAAAAGCATTATACTTGGACAATCAAT**TTTA**A | AAUUUCUACUGUUGUAGAUAUAAGCGCUUUGUCAUG |
|  |  |  | plcR-R | Ditto |  |
| plcR-3 | TACGAATAAGCGCTTTGTCAT | TTTG | plcR-3F | ATGCAAAAGCATTATACTTGGACAATCAA**TTTG**AA | AAUUUCUACUGUUGUAGAUAAUAAGCGCUUUGUCAU |
|  |  |  | plcR-R | Ditto |  |
| plcR-4 | ATACGAATAAGCGCTTTGTCA | TTTC | plcR-4F | ATGCAAAAGCATTATACTTGGACAATCATTTCGAA | AAUUUCUACUGUUGUAGAUGAAUAAGCGCUUUGUCA |
|  |  |  | plcR-R | Ditto |  |
| plcR-5 | AATACGAATAAGCGCTTTGTC | TTTA | plcR-5F | ATGCAAAAGCATTATACTTGGACAATC**TTTA**CGAA | AAUUUCUACUGUUGUAGAUCGAAUAAGCGCUUUGUC |
|  |  |  | plcR-R | Ditto |  |
| plcR-6 | CAATACGAATAAGCGCTTTGT | TTTT | plcR-6F | ATGCAAAAGCATTATACTTGGACAAT**TTTT**ACGAA | AAUUUCUACUGUUGUAGAUACGAAUAAGCGCUUUGU |
|  |  |  | plcR-R | Ditto |  |
| plcR-R1 | GCTTATTCGTATTGATTGTCC | TTTT | plcR-R1F | AGCTTTATTTGCATGACAAAGC**TTTT** | AAUUUCUACUGUUGUAGAUAUUCGUAUUGAUUGUCC |
|  |  |  | plcR-RR | GAGTTTGATGTGAAGGTGAGACATAATCATGC |  |
| plcR-R2 | CGCTTATTCGTATTGATTGTC | TTTT | plcR-R2F | AGCTTTATTTGCATGACAAAG**TTTT**T | AAUUUCUACUGUUGUAGAUUAUUCGUAUUGAUUGUC |
|  |  |  | plcR-RR | Ditto |  |
| plcR-R3 | GCGCTTATTCGTATTGATTGT | TTTC | plcR-R3F | AGCTTTATTTGCATGACAAA**TTTC**TT | AAUUUCUACUGUUGUAGAUUUAUUCGUAUUGAUUGU |
|  |  |  | plcR-RR | Ditto |  |
| plcR-R4 | AGCGCTTATTCGTATTGATTG | TTTG | plcR-R4F | AGCTTTATTTGCATGACAA**TTTG**CTT | AAUUUCUACUGUUGUAGAUCUUAUUCGUAUUGAUUG |
|  |  |  | plcR-RR | Ditto |  |
| plcR-R5 | AAGCGCTTATTCGTATTGATT | TTTC | plcR-R5F | AGCTTTATTTGCATGACA**TTTC**GCTT | AAUUUCUACUGUUGUAGAUGCUUAUUCGUAUUGAUU |
|  |  |  | plcR-RR | Ditto |  |
| plcR-R6 | AAAGCGCTTATTCGTATTGAT | TTTG | plcR-R6F | AGCTTTATTTGCATGAC**TTTG**CGCTT | AAUUUCUACUGUUGUAGAUCGCUUAUUCGUAUUGAU |
|  |  |  | plcR-RR | Ditto |  |
| gyrA-1 | GATTCAGCTGTTTATGAAACG | TTTT | gyrA-1F | AGTAATCGGTAAGTATCACCCTCATGGT**TTTT** | AAUUUCUACUGUUGUAGAUCAGCUGUUUAUGAAACG |
|  |  |  | gyrA-R | CGCTGAATCTCCATCGACAGATCC |  |
| gyrA-2 | TGATTCAGCTGTTTATGAAAC | TTTT | gyrA-2F | AGTAATCGGTAAGTATCACCCTCATGG**TTTT**T | AAUUUCUACUGUUGUAGAUUCAGCUGUUUAUGAAAC |
|  |  |  | gyrA-R | Ditto |  |
| gyrA-3 | GTGATTCAGCTGTTTATGAAA | TTTA | gyrA-3F | AGTAATCGGTAAGTATCACCCTCATG**TTTA**TT | AAUUUCUACUGUUGUAGAUUUCAGCUGUUUAUGAAA |
|  |  |  | gyrA-R | Ditto |  |
| gyrA-4 | GGTGATTCAGCTGTTTATGAA | TTTG | gyrA-4F | AGTAATCGGTAAGTATCACCCTCAT**TTTG**ATT | AAUUUCUACUGUUGUAGAUAUUCAGCUGUUUAUGAA |
|  |  |  | gyrA-R | Ditto |  |
| gyrA-5 | TGGTGATTCAGCTGTTTATGA | TTTT | gyrA-5F | AGTAATCGGTAAGTATCACCCTCA**TTTT**GATT | AAUUUCUACUGUUGUAGAUGAUUCAGCUGUUUAUGA |
|  |  |  | gyrA-R | Ditto |  |
| gyrA-6 | ATGGTGATTCAGCTGTTTATG | TTTG | gyrA-6F | AGTAATCGGTAAGTATCACCCTC**TTTG**TGATT | AAUUUCUACUGUUGUAGAUUGAUUCAGCUGUUUAUG |
|  |  |  | gyrA-R | Ditto |  |
| gyrA-R1 | AGCTGAATCACCATGAGGGTG | TTTT | gyrA-R1F | CCATACGTACCATCGTTTCATAAAC**TTTT** | AAUUUCUACUGUUGUAGAUGAAUCACCAUGAGGGUG |
|  |  |  | gyrA-RR | ATCGTAGGGTTTTATATGCGATGAATGATTTAGG |  |
| gyrA-R2 | CAGCTGAATCACCATGAGGGT | TTTC | gyrA-R2F | CCATACGTACCATCGTTTCATAAA**TTTC**T | AAUUUCUACUGUUGUAGAUUGAAUCACCAUGAGGGU |
|  |  |  | gyrA-RR | Ditto |  |
| gyrA-R3 | ACAGCTGAATCACCATGAGGG | TTTG | gyrA-R3F | CCATACGTACCATCGTTTCATAA**TTTG**CT | AAUUUCUACUGUUGUAGAUCUGAAUCACCAUGAGGG |
|  |  |  | gyrA-RR | Ditto |  |
| gyrA-R4 | AACAGCTGAATCACCATGAGG | TTTA | gyrA-R4F | CCATACGTACCATCGTTTCATA**TTTA**GCT | AAUUUCUACUGUUGUAGAUGCUGAAUCACCAUGAGG |
|  |  |  | gyrA-RR | Ditto |  |
| gyrA-R5 | AAACAGCTGAATCACCATGAG | TTTC | gyrA-R5F | CCATACGTACCATCGTTTCAT**TTTC**AGCT | AAUUUCUACUGUUGUAGAUAGCUGAAUCACCAUGAG |
|  |  |  | gyrA-RR | Ditto |  |
| gyrA-R6 | TAAACAGCTGAATCACCATGA | TTTA | gyrA-R6F | CCATACGTACCATCGTTTCA**TTTA**CAGCT | AAUUUCUACUGUUGUAGAUCAGCUGAAUCACCAUGA |
|  |  |  | gyrA-RR | Ditto |  |
| parC-1 | GATTCCTCTGTATATGAAGCG | TTTT | parC-1F | GGTAACTATCACCCGCACGGTTTTT | AAUUUCUACUGUUGUAGAUCCUCUGUAUAUGAAGCG |
|  |  |  | parC-R | CAACACTACCATTATTACCATGCATCTCAAC |  |
| parC-2 | TGATTCCTCTGTATATGAAGC | TTTT | parC-2F | GGTAACTATCACCCGCACGGTTTTT | AAUUUCUACUGUUGUAGAUUCCUCUGUAUAUGAAGC |
|  |  |  | parC-R | Ditto |  |
| parC-3 | GTGATTCCTCTGTATATGAAG | TTTA | parC-3F | GGTAACTATCACCCGCACGTTTATT | AAUUUCUACUGUUGUAGAUUUCCUCUGUAUAUGAAG |
|  |  |  | parC-R | Ditto |  |
| parC-4 | GGTGATTCCTCTGTATATGAA | TTTG | parC-4F | GGTAACTATCACCCGCACTTTGATT | AAUUUCUACUGUUGUAGAUAUUCCUCUGUAUAUGAA |
|  |  |  | parC-R | Ditto |  |
| parC-5 | CGGTGATTCCTCTGTATATGA | TTTT | parC-5F | GGTAACTATCACCCGCATTTTGATT | AAUUUCUACUGUUGUAGAUGAUUCCUCUGUAUAUGA |
|  |  |  | parC-R | Ditto |  |
| parC-6 | ACGGTGATTCCTCTGTATATG | TTTG | parC-6F | GGTAACTATCACCCGCTTTGTGATT | AAUUUCUACUGUUGUAGAUUGAUUCCUCUGUAUAUG |
|  |  |  | parC-R | Ditto |  |
| parC-R1 | AGAGGAATCACCGTGCGGGTG | TTTG | parC-R1F | GACTTAAACGTACCATCGCTTCATATACTTTG | AAUUUCUACUGUUGUAGAUGAAUCACCGUGCGGGUG |
|  |  |  | parC-RR | ACGGCTTAAAACCAGTACAAAGACG |  |
| parC-R2 | CAGAGGAATCACCGTGCGGGT | TTTA | parC-R2F | GACTTAAACGTACCATCGCTTCATATATTTAG | AAUUUCUACUGUUGUAGAUGGAAUCACCGUGCGGGU |
|  |  |  | parC-RR | Ditto |  |
| parC-R3 | ACAGAGGAATCACCGTGCGGG | TTTG | parC-R3F | GACTTAAACGTACCATCGCTTCATATTTTGAG | AAUUUCUACUGUUGUAGAUAGGAAUCACCGUGCGGG |
|  |  |  | parC-RR | Ditto |  |
| parC-R4 | TACAGAGGAATCACCGTGCGG | TTTA | parC-R4F | GACTTAAACGTACCATCGCTTCATATTTAGAG | AAUUUCUACUGUUGUAGAUGAGGAAUCACCGUGCGG |
|  |  |  | parC-RR | Ditto |  |
| parC-R5 | ATACAGAGGAATCACCGTGCG | TTTC | parC-R5F | GACTTAAACGTACCATCGCTTCATTTTCAGAG | AAUUUCUACUGUUGUAGAUAGAGGAAUCACCGUGCG |
|  |  |  | parC-RR | Ditto |  |
| parC-R6 | TATACAGAGGAATCACCGTGC | TTTA | parC-R6F | GACTTAAACGTACCATCGCTTCATTTACAGAG | AAUUUCUACUGUUGUAGAUCAGAGGAAUCACCGUGC |
|  |  |  | parC-RR | Ditto |  |

**Table S3 CRISPR-Cas12a reaction system constituents (20 μL**)

| Reagent | Volume (μL) |
| --- | --- |
| Nuclease-free water (Thermo Fisher Scientific, Waltham, MA,USA) | 12 |
| NEBuffer™ 3 (New England Biolabs,Ipswich, MA,USA) | 2 |
| LbCas12a nuclease (0.15mg/mL) | 1 |
| HEX-N12-BHQ1 Single-stranded DNA probe (10 μM) | 2 |
| crRNA (10 μM) | 1 |
| PCR products | 2 |

**Table S4 RPA amplification primers and reaction system**

| RPA amplification primers | |  | RPA reaction | |
| --- | --- | --- | --- | --- |
| Primer name | Primer sequences（5’-3’） |  | Reagent | Volume (μL) |
| RPA-plcR-F | GTTCAATAGCTTTATTTGCATGACTTTGCGCTT |  | Primer F(10μM) | 2.4 |
| RPA-plcR-R | AGAGTTTGATGTGAAGGTGAGACATAATCATGC |  | Primer R(10μM) | 2.4 |
| RPA-lef-F | GCATCAATATTTTGAATATCCCTTTTATACTGC |  | 2×Reaction Buffer | 25 |
| RPA-lef-R | CTTGATATTCAACCATATGATATTAATCAAAGG |  | dNTPs(2.5 mM each) | 9.2 |
| RPA-capB-F | CCGGATGAGCATTCAACATACCACGGAATGCTG |  | 10x Probe E-mix | 5 |
| RPA-capB-R | TTGATTACATGGTCTTCCCAGATAATGCATCGC |  | 20x Core Reaction Mix | 2.5 |
| RPA-CR5-F | GAGAAGTCAACGAGCACGACACCAGATTTTAA |  | bacterial genome | 2 |
| RPA-CR5-R | GTGAAGAAGAACTATTAGATGATTCTGTAGAAC |  | 280mM MgOAc | 2.5 |

**Table S5 qPCR amplification primers and TaqMan probe**

| TaqmanCR5-F | GCTTGTTGATTTCTCTTC |
| --- | --- |
| TaqmanCR5-R | GCAGAAATTAAAGCTGATG |
| CR5probeG | 5'-(FAM)ACTTATCTTCTGTGTTACTTTCTGGTG(MGB)-3' |
| CR5probeT | 5'-(VIC)ACTTATCTTCTGTTTTACTTTCTGGTG(MGB)-3' |
| TaqmanplcR-F^a^ | CCAATCAATGTCATACTATTAATTTGACAC |
| TaqmanplcR-R^a^ | ATGCAAAAGCATTATACTTGGACAAT |
| plcRprobeT^a^ | 5'-(FAM)CAAAGCGCTTATTCGTATT(MGB)-3' |
| plcRprobeG^a^ | 5'-(VIC)AAAGCGCTTCTTCGTATT(MGB)-3' |
| TaqmanLef-F | GATGCTGGCAAGATATGA |
| TaqmanLef-R | TGGCTCAATAGGAATCTG |
| Lefprobe | 5'-(FAM)AGCACTATCAACACTGGAGCGA(MGB)-3' |
| TaqmanCapB-F | TACCGTTACAAGACTAATTAC |
| TaqmanCapB-R | GTTGCTCACCGATATTAG |
| CapBprobe | 5'-(FAM)AATCGGTTGCTCCTCGTCAGTAA(MGB)-3' |

^a^ the *plcR* primers reference: Easterday W R, Ert M, Simonson TS, et al. Use of Single Nucleotide Polymorphisms in the plcR Gene for Specific Identification of Bacillus anthracis[J]. Journal of Clinical Microbiology, 2005, 43(4):1995-1997.
